# Dispersion of metallic/ceramic matrix nanocomposite material through porous surfaces in magnetized hybrid nanofluids flow with shape and size effects

**DOI:** 10.1038/s41598-021-91152-z

**Published:** 2021-06-10

**Authors:** M. Zubair Akbar Qureshi, S. Bilal, M. Y. Malik, Qadeer Raza, El-Sayed M. Sherif, Yong-Min Li

**Affiliations:** 1 Department of Mathematics, Air University Islamabad, Multan, 60000 Pakistan; 2 Department of Mathematics, P.A. F Complex, Sector E-9, Air University, Islamabad, 44000 Pakistan; 3 Department of Mathematics, College of Sciences, King Khalid University, Abha, 61413 Kingdom of Saudi Arabia; 4 Research Chair for Tribology, Surface, and Interface Sciences (TSIS), Department of Physics and Astronomy, College of Science, King Saud University, P.O. Box 2455, Riyadh, 11451 Saudi Arabia; 5 Center of Excellence for Research in Engineering Materials (CEREM), King Saud University, P.O. Box 800, Riyadh, 11421 Saudi Arabia; 6 Department of Mathematics, Huzhou University, Huzhou, 313000 People’s Republic of China

**Keywords:** Engineering, Materials science, Mathematics and computing

## Abstract

Matrix nanocomposites are high performance materials possessing unusual features along with unique design possibilities. Due to extraordinary thermophysical characteristic contained by these matrix nanocomposites materials they are useful in several areas ranging from packaging to biomedical applications. Being an environment friendly, utilization of nanocomposites offer new technological opportunities for several sectors of aerospace, automotive, electronics and biotechnology. In this regards, current pagination is devoted to analyze thermal features of viscous fluid flow between orthogonally rotating disks with inclusion of metallic matrix nanocomposite (MMNC) and ceramic matrix nanocomposites (CMNC) materials. Morphological aspects of these nanomaterials on flow and heat transfer characteristics has been investigated on hybrid viscous fluid flow. Mathematical structuring of problem along with empirical relations for nanocomposites materials are formulated in the form of partial differential equations and later on converted into ordinary differential expressions by using suitable variables. Solution of constructed coupled differential system is found by collaboration of Runge–Kutta and shooting methods. Variation in skin friction coefficient at lower and upper walls of disks along with measurement about heat transfer rate are calculated against governing physical parameters. Impact of flow concerning variables on axial, radial components of velocity and temperature distribution are also evaluated. Contour plots are also drawn to explore heat and thermal profiles. Comparison and critical analysis of MMNc and CMNc have been presented at lower and upper porous disks. Our computed analysis indicates that hybrid nanofluids show significant influence as compared to simple nanofluids with the permutation of the different shape factors.

## Introduction

With advancement in technology modern thermal management systems like central processing units in computers, light emitting diodes, transistor, solar collectors, audio amplifiers and so forth requires liquids with improved thermal conductivity. Since, ordinary fluids are unable to meet desired requirements for advanced industrial and technological procedures so insertion of nanoparticles in poorly conducting ordinary liquids are managed. The appropriate mixture of nanoparticles in specified ratios has generated exceptional thermal performance of weak thermalized systems. The concept of addition of nanoparticles was inaugurated by Choi and Eastman^1^ which laid the basis of nanotechnology. Initially, nanofluids are considered as single phase flows but it is experimentally proved that consideration of nanoliquid as two phase compositions is much more beneficial. Yamada et al.^2^ discussed the combined two or more than two organic and inorganic constituents at nanoscale which are obliged in polymers. This idea executed hybridization of nanoparticles with different organic/inorganic compositions. The hybridization of base fluid with multiply structured nanoparticles change dynamics of industrial world^3^.

From advent of twenty-first century a new findings in nanotechnology is discovered which is the concept of matrix nanocomposites materials. It is a two phase material with one phase consisting of solid material and other phase is nanometer sized particles. Nanomaterials are lightweight, highly strength full, sustainable, elevated temperature strengthened, corrosion resisted can be used in a wide variety of applications, e.g. aerospace, automotive, biomedical engineering etc. On basis of such mechanical, electrical, thermal, optical, electrochemical and catalytic features nanocomposites are characterized in to ceramic/metal matrix nanocomposites. MMNC is the material which composes of metal matrix distributed in concrete, ceramic, or organic substances. The reinforcement and distribution of matrix material into nano-scale particles which generates remarkable improvement in mechanical properties of formed composition. In metal matrix composites metals like copper, magnesium, and aluminum are capitalized as base metal. Since, base materials are metal which have stiffness and strength so MMNC is obliged in aviation, aerospace, defensive weapons and other areas. The most widely used reinforcements in matrix nanocomposites are Silicon Carbide (SiC), TiO_2_, Aluminum Oxide (Al_2_O_3_) because of fact that they provide strong wear resistance and compressive power. In addition, majority volume of matrix metal composites are filled by a ceramic such as nitrides, oxides, silicides and borides. To gain optimized usage and evoking of precise nanoscopic features the ceramics as well as metallic elements are uniformly distributed. Zeeshan et al.^4^ investigated influence of morphological aspects of nanoparticles by varying shape on features of fluid floating across a spinning disk. Ahmad et al.^5^ depicted magnetized nano squeezed flow between two parallel disks along with consideration of viscous dissipation and multi natured hybrid particles. Haq et al.^6^ inserted Manganese-Zinc ferrite and Cobalt ferrite in viscous fluid flow between parallel disks and adumbrated enhancement in thermal conductance. Heat transfer features by inducting platelet, cylinder, and brick-shaped copper nanoparticles in water was studied by Khan et al.^7^. Hajabdollahi et al.^8^ examined morphometric aspects of (Al_2_O_3_) nanoparticles by considering platelets, spherical, cylindrical shapes, and bricks shapes on performance of tube heat exchangers. Kucharik et al.^9^ discussed absorption of metallic particles in water for formation of colloidal substances used in heat transfer diffusion processes in lasers. Ghozatloo et al.^10^ analyzed experimentally about dynamics of insertion of gold nanoparticles in water and ethylene glycol and build comparative framework regarding affectivity of particles against viscosity variant base liquids.

Heat is form of energy that arises due to temperature difference. Heat transfer with in flow procedures has received several applications like in electric power production, vehicle propulsion and climate control devices, home heating, and cooling appliances and so many. Heat transmits during the flow by the way of conduction, convection, and radiation. Generally, heat transfer in fluid arises due to convective motion of liquid molecules and enhanced is done by uplifting thermal conductivity of liquid. Researchers have experimentally discovered that this is executed by adding nanoparticles. This experimentation is proved to be successful when cooling and heating of domestic appliance like refrigerator, coils and electrical device is raised by nanoparticles inclusion. Chamkha et al.^11^ investigated unsteady squeezing flow and upsurge in convective motion of fluid molecules bounded between two parallel disks. Mochizuki et al.^12^ conducted experimental analysis the mount in thermal conductivity of viscous fluid flowing between two parallel heated disks rotated radially. Characteristics of hall current on hybridized nanoliquid flow across spinning disk was demonstrated by Acharya et al.^13^. Bhattacharyya et al.^14^ examined enhancement in heat transfer properties of viscous liquid between two coaxial disks by adding carbon nanotubes. Bhat et al.^15^ analyzed heat transfer in flow through addition of nanostructures in a porous disk by employing tangential slip level constraint at boundary. Maskeen et al.^16^ established hybridized nano fluid flow with combination of alumina and copper particles under impact of Lorentz magnetic field. Ghaffar et al.^17^ numerically studied heat transfer in incompressible flow of fluid between two orthogonally passing coaxial porous disks with interaction of ferromagnetic nanoparticles.

In recent years some inconsistencies in reported results regarding heat transfer rate with addition of nanoparticles is found. So, researchers have tried to use hybridized nanofluid which is engineered suspension of dissimilar nanoparticles in mixture or in composite form. This idea has further improved heat transfer and pressure drop characteristics and removed disadvantages of individual suspension. With the formation of hybridized nanofluids a better thermal network containing synergistic effect of nanomaterials is attained. In this regard several studies is available in literature like Behnam et al.^18^ used hybridization of Titanium dioxide with Silicon Carbide in heavy water limited between moveable disks under effect of vertical magnetic field. Use of hybrid nanofluid during treatment of cancer patients was disclosed by Hayat et al.^19^. Enhancement in thermal transfer by using hybrid nanoliquid during industrial procedures was explained by Hussein et al.^20^. Devi et al.^21^ examined hydromagnetic hybrid nano-liquid (Cu-Al_2_O_3_/water) for raise in temperature and heat transfer up gradation in liquid flowing in permeable flow domain. Sarkar et al.^22^ evaluated optimistic impact of hybridization of nanoparticles for enhancing temperature profile. Sundar et al.^23^ provided comparative analysis regarding heat transfer features attained with addition of ordinary and hybridized nanoparticles.

Magneto hydrodynamics describe flow behavior of moving conducting liquid which in turns polarized it. Impact of magnetic field is evaluated in industrial processes like fuel industry, electric generators, nuclear plants, aerodynamics, crystal production and so forth. Tamim et al.^24^ analyzed MHD flow and its practical appliance in multiple sectors. The field of magneto hydrodynamics was introduced by Alfven et al.^25^. Emad et al.^26^ demonstrated analytical and numerical treatment about hybrid magnetic nano material in porous stretching/shrinking medium. Ghadikolaei et al.^27^ analyzed heat transfer features of electrical conducting hybridized nano liquid (TiO_2_-Cu) in water by varying shape of nanoparticles. Hayat et al.^28^ explored magnetically effected fluid flowing between two parallel spinning disks by using analytical approach. Ahmad et al.^29^ explored impact of magnetic field on asymmetric flow of fluid and found decrease in momentum profile. Rashidi et al.^30^ magnetic flow of viscous fluid by interpreting impact of it on thermal conductivity. Raddya et al.^31^ explored impact of magnetic field on nano-liquid flow in rotating frame and found reduction in velocity against Hartmann number. Mliki et al.^32^ demonstrated convective flow of nano fluid under appliance of MHD in an enclosure by examining thermo physical features. Turkyilmazoglu et al.^33^ explicated heat transfer features of magnetized viscous fluid flow generated by spinning disks. Some recent literature about flow characteristics of fluid under appliance of magnetic field in different physical configurations is enclosed in^34–38^. Sheikholeslami et al.^39^ aimed to investigate hot gas flow inside inner pipe and operating fluid in the annulus region filled with nanoparticles composed of CuO. Wang et al.^40^ analyzed utilization of hybrid nanofluid in solar photovoltaic system using the spectral beam splitting technology. Chuanpan et al.^41^ described a high-efficiency sensing system working with carbon nanofibers and discussed it efficiency by varying shape and amount of fibers. Guo et al.^42^ founded application hybridized nano based materials in environmental protection. Yun et al.^43^ determined degradation performance of nano-TiO_2_ as a coating material used in roads and bridge construction. Sheikholeslami & Farshad^44^ discussed the solar collector system with turbulater effect for hybrid nanoparticles. Hybrid nanofluid is useful to increase the exergy efficiency of solar system and produces higher overall efficiency^45^.

To the best of the author’s knowledge there has been a scarcity in research regarding analysis of morphology effect of hybrid nanofluids coupled with matrix metal and ceramic matrix nanocomposites on thermo physical features of fluid flow between two orthogonally placed porous disks. Mathematical modeling in the form of partial differential equations is developed by considering shape and size of different (MMNC) and (CMNC). Later on, attained PDE’s are converted into ODE’s by using appropriate variables. Solution of intricate coupled differential system is attained by implementing Runge–Kutta and shooting method jointly. Impact of involved dimensionless parameters on associated profiles is adorned.

## Mathematical formulation

**Figure 1:**
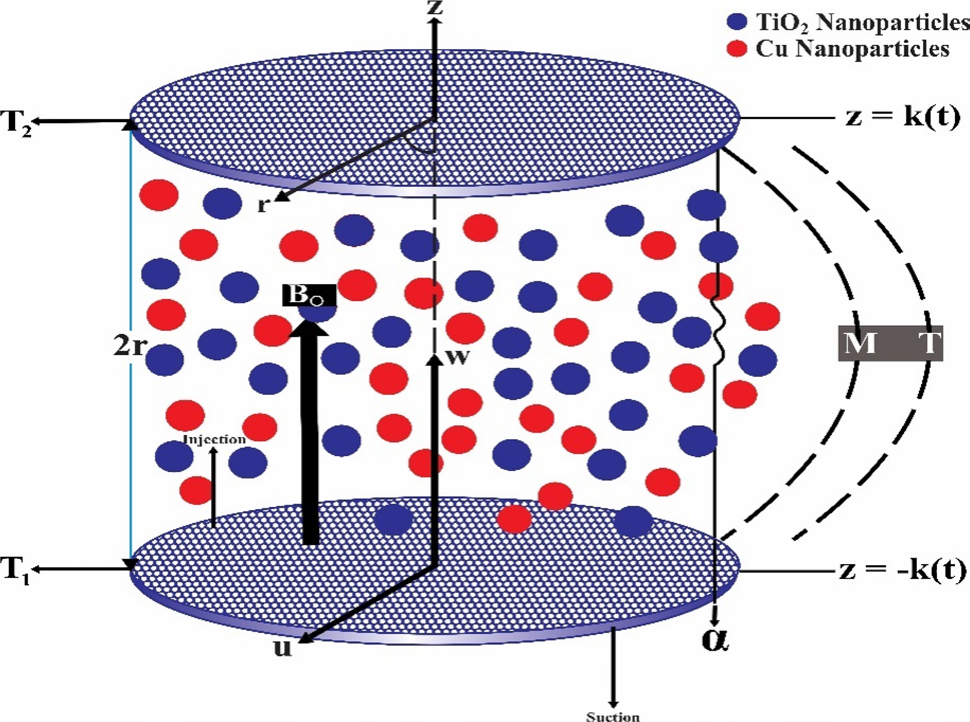
Physical model.

Let us consider an incompressible, viscous, laminar, unsteady, 2D flow of hybrid nanofluid containing TiO_2_-Cu/water nanoparticles between two orthogonally moving porous coaxial disk in the presence of the external magnetic field applied in z-direction. The diameter of boundary disks is 2r. The physical model takes in a cylindrical coordinate system $\left( {r,\theta ,z} \right)$ but velocity v disappears and u and w part of the velocity in the lines of r and z, respectively. The temperature at the lower disk is denoted by $T_{1}$ and the upper disk is $T_{2}$ show in Fig. 1.

Law of conservation of mass, momentum, and energy are as follows referred to Kashif et al.^46^

1 $$\frac{\partial u}{{\partial r}} + \frac{u}{r} + \frac{\partial w}{{\partial z}} = 0$$

2 $$\frac{\partial u}{{\partial t}} + {\text{u}}\frac{\partial u}{{\partial r}} + {\text{w}}\frac{\partial u}{{\partial z}} = - \frac{1}{{\rho_{hnf} }}\frac{\partial p}{{\partial r}} + \upsilon_{hnf} \left( {\frac{{\partial^{2} u}}{{\partial r^{2} }} + \frac{1}{r}\frac{\partial u}{{\partial r}} - \frac{u}{{r^{2} }} + \frac{{\partial^{2} u}}{{\partial z^{2} }}} \right) - \frac{{\sigma_{e} B_{0}^{2} }}{{\rho_{hnf} }}{\text{u}}$$

3 $$\frac{\partial w}{{\partial t}} + {\text{u}}\frac{\partial w}{{\partial r}} + {\text{w}}\frac{\partial w}{{\partial z}} = - \frac{1}{{\rho_{hnf} }}\frac{\partial p}{{\partial z}} + \upsilon_{hnf} \left( {\frac{{\partial^{2} w}}{{\partial r^{2} }} + \frac{1}{r}\frac{\partial w}{{\partial r}} + \frac{{\partial^{2} w}}{{\partial z^{2} }}} \right)$$

4 $$\frac{\partial T}{{\partial t}} + {\text{u}}\frac{\partial T}{{\partial r}} + {\text{w}}\frac{\partial T}{{\partial z}} = \alpha_{hnf} \frac{{\partial^{2} T}}{{\partial z^{2} }}$$

where $\rho_{hnf}$ is the density of ($HN_{fd}$), $\sigma_{e}$ is the electrical conductivity, $B_{0}^{2} {\text{ of the}} {\text{magnetic field is the strength}}, p {\text{is the}}$ pressure and T is the temperature, $\alpha_{hnf}$ is the thermal diffusivity,$\upsilon_{hnf}$ is the kinematics viscosity of the (*HN*_*fd*_).

### Thermophysical Properties of hybrid nanofluid referred to^47–49^

Thermophysical properties of ($N_{fd}$) convectional hybrid $\left( {{\text{TiO}}_{2} - {\text{Cu}}/{\text{H}}_{2} {\text{O}}} \right)$ then $\varphi_{1 = } \varphi_{{{\text{TiO}}_{{2}} }}$, $\varphi_{2 = } \varphi_{{{\text{Cu}}}}$, $\rho_{s1} = \rho_{{{\text{TiO}}_{{2}} }}$, $\rho_{s2} = \rho_{{{\text{Cu}}}}$, $\left( {\rho c_{p} } \right)_{s1} = \left( {\rho c_{p} } \right)_{{{\text{TiO}}_{{2}} }}$, $\left( {\rho c_{p} } \right)_{s2} = \left( {\rho c_{p} } \right)_{{{\text{Cu}}}}$, $k_{s1} = k_{{{\text{TiO}}_{{2}} }}$ and $k_{s2} = k_{{{\text{Cu}}}}$.

#### Effective dynamic viscosity

Efsa et al.^48^ delivered a theory of diameter depending viscosity

5 $$\mu_{hnf} = \mu_{bf} (1 + 0.1008((\varphi_{1} )^{0.69574} \left( {dp_{1} )^{0.44708} + (\varphi_{2} )^{0.69574} \left( {dp_{2} } \right)^{0.44708} } \right)$$

#### Effective density

The effective density of *HN*_*fd*_ is denoted by the $\rho_{hnf}$

6 $$\rho_{hnf} = \varphi_{1} \rho_{s1} + \varphi_{2} \rho_{s2} - \left( {1 - \varphi_{1} - \varphi_{2} } \right) \rho_{bf}$$

where $\varphi_{1} \;{\text{and }}\;\varphi_{2}$ shows volume fraction, $\rho_{bf}$ represent the density of base fluid, $\rho_{s1}$ or $\rho_{s2}$ indicates the density of solid NP’s, $\rho_{hnf}$ the density of hybrid nanofluid.

#### Effective heat capacitance

Hybrid nanofluid effective heat capacitance is denoted by

7 $$\rho_{hnf} = \varphi_{1} \left( {\rho c_{p} } \right)_{s1} + \varphi_{2} (\rho c_{p} )_{s2} - \left( {1 - \varphi_{1} - \varphi_{2} } \right) \left( {\rho c_{p} } \right)_{bf}$$

where $\left( {\rho c_{p} } \right)_{s1}$ and $(\rho c_{p} )_{s2}$ specify the thermal capacitance for strong NP’s and the thermal capacitance for (*B*_*fd*_) is defined in $\left( {\rho c_{p} } \right)_{bf}$.

#### Effective thermal conductivity

The thermal conductivity of hybrid nano fluid of specific size and shape factor of NP’s is described as under

8 $$k_{hnf} = \frac{{k_{s2} + \left( {n_{2} - 1} \right)k_{mbf} - \left( {n_{2} - 1} \right)\varphi_{2} \left( {k_{mbf} - k_{s2} } \right)}}{{k_{s2} + \left( {n_{2} - 1} \right)km_{bf} + \varphi_{2} \left( {km_{bf} - k_{s2} } \right)}}k_{mbf}$$

where

$$k_{mbf} = \frac{{k_{s1} + \left( {n_{1} - 1} \right)k_{f} - \left( {n_{1} - 1} \right)\varphi_{1} \left( {k_{f} - k_{s1} } \right)}}{{k_{s1} + \left( {n_{1} - 1} \right)k_{f} + \varphi_{1} \left( {k_{f} - k_{s1} } \right)}}k_{f}$$

where $k_{hnf}$ is thermal conductivity for (*HN*_*fd*_), $k_{s1}$ and $k_{s2}$ are thermal conductivity of solid nanoparticles,$n_{1}$ *and* $n_{2}$ are different size factors (spherical, bricks, plates, cylindrical), $k_{bf}$ and $k_{mbf}$ are thermal conductivity and shapes factor base fluid. Furthermore, different shape of first (Fe_3_O_4_), second (Al_2_O_3_), third (TiO_2_) and fourth (Cu) NP’s (*n*_1_ and *n*_2_) based on thermal conductivity of (*HN*_*fd*_). The thermal conductivity of copper nanoparticles is high than titanium oxide.

9 $$\upsilon_{hnf} = \frac{{\mu_{hnf} }}{{\rho_{hnf} }}\;{\text{and}}\; \alpha_{hnf} = \frac{{k_{hnf} }}{{(\rho c_{p} )_{hnf} { }}},\quad Pr = \frac{{\left( {\mu C_{p} } \right)_{bf} }}{{k_{bf} }}$$

where $\rho_{s} \;{\text{and}}\; \rho_{f}$ are density for solid and fluid fractions, the specific capacitance for (*HN*_*fd*_) $(\rho c_{p} )_{hnf} ,$ the ($HN_{fd}$) for the effect of thermal conductivity $k_{hnf}$.

For boundary situations the flow state is:

10 $$\begin{aligned} & z = - k(t)\;{\text{u}} = 0,{\text{w}} = - A_{1} k^{\prime}\left( t \right)\;{\text{at}}\;T = T_{1} \;{\text{and}}\;z = k,{\text{u}} = 0 \\ & {\text{w}} = A_{1} k^{\prime}\left( t \right)\;{\text{at}}\;T = T_{2} \\ \end{aligned}$$

Here, $A_{1}$ is the metric of penetrability of the partition, and the dot represents the w.r.t time t derivative.

Following similarity variables are used

11 $$\eta = \frac{z}{k}\quad {\text{u}} = - \frac{{r\upsilon_{f} }}{{k^{2} }}F_{\eta } \left( {\eta ,t} \right)\quad {\text{w}} = \frac{{2\upsilon_{f} }}{k}F\left( {\eta ,t} \right) \quad \theta = \frac{{T - T_{2} }}{{T_{1} - T_{2} }}$$

and result

12 $$\frac{{\upsilon_{hnf} }}{{\upsilon_{f} }}F_{\eta \eta \eta \eta } + \alpha (3F_{\eta \eta } + \eta F_{\eta \eta \eta } ) - 2FF_{\eta \eta \eta } - \frac{{k^{2} }}{{\upsilon_{f} }}F_{\eta \eta t} - \frac{{\rho_{f} }}{{\rho_{hnf} }} + MF_{\eta \eta } = 0$$

13 $$\theta_{\eta \eta } + \frac{{\upsilon_{f} }}{{\alpha_{hnf} }}(\alpha \eta - 2F)\theta_{\eta } - \frac{{k^{2} }}{{\alpha_{hnf} }}\theta_{t} = 0$$

Associated boundary conditions

14 $$\begin{aligned} & \eta = - 1, F = - {\varvec{R}},F_{\eta } = 0,\theta = 1, \\ & {\text{and}}\; \eta = 1, F = R,F_{\eta } = 0,\theta = 0, \\ \end{aligned}$$

Here, α = $\frac{{kk^{\prime}\left( t \right)}}{{\upsilon_{f} }}$ is the wall expansion ratio, *R* = $\frac{{A_{1} kk^{\prime}\left( t \right)}}{{2\upsilon_{f} }}$ is the absorptivity Reynolds number, Sc = $\frac{{\upsilon_{f} }}{D}$ is the Schmidt number and M = $\frac{{\sigma_{e} B_{0}^{2} k^{2} }}{{\mu_{f} }}$ is the magnetic parameter.

Finally, we set F = $f$ *R*, and consider the case following Majdalani et al.^50^ when α is a constant, $f$ = $f$ (η) and θ = θ (η), which leads to $\theta_{t}$ = 0 and $f_{\eta \eta t}$ = 0.Thus we have the following equations

15 $$\frac{{\upsilon_{hnf} }}{{\upsilon_{f} }}f_{\eta \eta \eta \eta } + \alpha (3f_{\eta \eta } + \eta f_{\eta \eta \eta } ) - 2Rff_{\eta \eta \eta } - \frac{{\rho_{f} }}{{\rho_{hnf} }}Mf_{\eta \eta } = 0$$

16 $$\theta_{\eta \eta } + \left( {\left( {1 - \left( {\varphi_{1} + \varphi_{2} } \right)} \right) + \left( {\varphi_{1} } \right)\left( {\frac{{\rho_{{cps_{1} }} }}{{\rho_{cpbf} }}} \right) + \left( {\varphi_{2} } \right)\left( {\frac{{\rho_{{cps_{2} }} }}{{\rho_{cpbf} }}} \right)} \right)\frac{{{ }k_{mbf} { }}}{{{ }k_{hnf} { }}}\frac{{{ }k_{bf} { }}}{{{ }k_{mbf} { }}}Pr(\alpha \eta - 2Rf)\theta_{\eta } = 0$$

Boundary conditions at lower and upper wall of channel

17 $$\eta \, = \, - {1};f = - 1,f_{\eta } = 0,\theta = 1\;{\text{and}}\;\eta = 1;f = 1,f_{\eta } = 0,\theta = 0$$

### Quantities of engineering interest

Skin friction and Nusselt number at both porous walls are computed coefficients which are of engineering interest are computed in this section.

#### Skin friction coefficients

The $C_{f1}$ and $C_{f - 1}$ are the representation of skin friction coefficient of lower and upper disk which is expressed as

18 $$C_{f - 1} = \frac{{\xi_{zr} |_{{{\eta } = { } - 1}} }}{{\rho_{f} \left( {k^{\prime}A_{1} } \right)^{2} }} = \frac{{(1 + 0.1008((\varphi_{1} )^{0.69574} \left( {dp_{1} )^{0.44708} + (\varphi_{2} )^{0.69574} \left( {dp_{2} } \right)^{0.44708} } \right)}}{{{\varvec{R}}_{{\varvec{r}}} }}f^{\prime\prime}\left( { - 1} \right),$$

$$C_{f1} = \frac{{\xi_{zr} |_{{{\eta } = { }1}} }}{{\rho_{f} \left( {k^{\prime}A_{1} } \right)^{2} }} = \frac{{(1 + 0.1008((\varphi_{1} )^{0.69574} \left( {dp_{1} )^{0.44708} + (\varphi_{2} )^{0.69574} \left( {dp_{2} } \right)^{0.44708} } \right)}}{{{\varvec{R}}_{{\varvec{r}}} }}f^{\prime\prime}\left( 1 \right).$$

where $R_{r} = 4\left( \frac{k}{r} \right)\left( \frac{1}{R} \right)^{2}$ stand for local Reynolds number and $\xi_{zr}$ are shear stresses at the lower and upper disk in the radial direction, respectively,

$$\begin{aligned} \xi_{zr} & = \mu_{hnf} \left( {\frac{\partial u}{{\partial z}}} \right)|_{{{\eta } = { } - 1}} = \mu_{bf} (1 + 0.1008((\varphi_{1} )^{0.69574} \left( {dp_{1} )^{0.44708} + (\varphi_{2} )^{0.69574} \left( {dp_{2} } \right)^{0.44708} } \right)\left( {\frac{{r\upsilon_{f} }}{{k^{3} }}} \right)f^{\prime\prime}\left( { - 1} \right) \\ \xi_{zr} & = \mu_{hnf} \left( {\frac{\partial u}{{\partial z}}} \right)|_{{{\eta } = { }1}} = \mu_{bf} (1 + 0.1008((\varphi_{1} )^{0.69574} \left( {dp_{1} )^{0.44708} + (\varphi_{2} )^{0.69574} \left( {dp_{2} } \right)^{0.44708} } \right)\left( {\frac{{r\upsilon_{f} }}{{k^{3} }}} \right)f^{\prime\prime}\left( 1 \right) \\ \end{aligned}$$

#### Nusselt number

The calculation at the lower and upper disk for heat transfer rate (Nusselt numbers) $Nu|_{{{\eta } = { } - 1}}$ and $Nu|_{{{\eta } = { }1}}$ are given as

19 $$\begin{aligned} & Nu|_{{{\eta } = { } - 1}} = \frac{{ks_{z} }}{{\kappa_{f} \left( {T_{1} - T_{2} } \right)}}|_{{{\eta } = { } - 1}} = - \frac{{k_{hnf} }}{{k_{f} { }}}\theta^{\prime}\left( { - 1} \right) \\ & Nu|_{{{\eta } = { }1}} = \frac{{ks_{z} }}{{\kappa_{f} \left( {T_{1} - T_{2} } \right)}}|_{{{\eta } = { }1}} = - \frac{{k_{hnf} }}{{k_{f} { }}}\theta^{\prime}\left( 1 \right) \\ \end{aligned}$$

where here heat flux is denoted as $s_{z}$ which is follows as,

$$\begin{aligned} & s_{z} |_{{{\eta } = { } - 1}} = - k_{hnf} \left( {\frac{\partial T}{{\partial z}}} \right)|_{{{\eta } = { } - 1}} = - \frac{{\left( {T_{1} - T_{2} } \right)}}{k}k_{hnf} \theta^{\prime}\left( { - 1} \right) \\ & s_{z} |_{{{\eta } = { }1}} = - k_{hnf} \left( {\frac{\partial T}{{\partial z}}} \right)|_{{{\eta } = { }1}} = - \frac{{\left( {T_{1} - T_{2} } \right)}}{k}k_{hnf} \theta^{\prime}\left( 1 \right) \\ \end{aligned}$$

where $${\varvec{R}} = \frac{{A_{1} kk^{\prime}\left( t \right)}}{{2\upsilon_{f} }}$$.

### Numerical procedure

Since attained couple system of ODEs manipulated in Eqs. (28) and (29) are intricate in nature and it is boundary value conditions so solution is found numerically instead of analytical approaches. For numerical computations shooting method in conjunction with Runge–Kutta method is implemented. Due to low computation, stability and accurate results in less time, Runge–Kutta method is better choice. The main advantage of this method is speed (computational cost) and additivity of the method with the initial value problem. Because of the significant applications of initial value problems into the real world/practical applications that’s why, to find the initial value problem with an appropriate shooting method is very successful.

#### Validation of the proposed method

In a shooting method, the missing initial condition at the initial point of the Interval is assumed, and the DE is then integrated numerically as an initial value problem. The accuracy of the assumed missing initial condition is then checked by comparing the calculated value of the dependent variable at the terminal point with its given value here. If a difference exist, another value of the missing initial condition must be assumed and the process is repeated. This process is continued until the agreement between the calculated and the given condition. For this purpose, given below Table 10 shows the convergence of our numerical results as the decrease in step-size gives us confidence on our computational procedure. Clearly, our boundary conditions satisfied with symmetric and accurate results of shear stress at lower wall as well.

An extensive representation of non-linear coupled system of ordinary differential equations along with coefficients possessing features of matrix composite materials and hybrid nano fluid

20 $$\begin{aligned} & \left( {\frac{{(1 + 0.1008((\varphi_{1} )^{0.69574} \left( {dp_{1} )^{0.44708} + (\varphi_{2} )^{0.69574} \left( {dp_{2} } \right)^{0.44708} } \right)}}{{\left( {\left( {1 - \left( {\varphi_{1} + \varphi_{2} } \right)} \right) + (\varphi_{1} )\left( {\frac{{\rho_{{s_{1} }} }}{{\rho_{bf} }}} \right) + \left( {\varphi_{2} } \right)\left( {\frac{{\rho_{{s_{2} }} }}{{\rho_{bf} }}} \right)} \right)}}} \right)f^{\prime\prime\prime\prime}\left[ \eta \right] - \alpha (3f^{\prime\prime}\left[ \eta \right] \\ & \quad + \eta f^{\prime\prime\prime}\left[ \eta \right]) - 2Rf\left[ \eta \right]f^{\prime\prime\prime}\left[ \eta \right] - \left( {\frac{1}{{\left( {\left( {1 - \varphi_{1} - \varphi_{2} } \right) + \varphi_{1} \left( {\frac{{\rho_{{s_{1} }} }}{{\rho_{bf} }}} \right) + \varphi_{2} \left( {\frac{{\rho_{{s_{2} }} }}{{\rho_{bf} }}} \right)} \right)}}} \right)M f^{\prime\prime}[\eta ] = 0 \end{aligned}$$

21 $$\begin{aligned} & \theta^{\prime\prime}\left[ \eta \right] + \left( {\left( {1 - \left( {\varphi_{1} + \varphi_{2} } \right)} \right) + \left( {\varphi_{1} } \right)\left( {\frac{{\rho_{{cps_{1} }} }}{{\rho_{cpbf} }}} \right) + \left( {\varphi_{2} } \right)\left( {\frac{{\rho_{{cps_{2} }} }}{{\rho_{cpbf} }}} \right)} \right) \\ & \left( {\frac{{k_{s2} + \left( {N - 1} \right)k_{mbf} + \varphi_{2} \left( {k_{mbf} - k_{s2} } \right)}}{{k_{s2} + \left( {N - 1} \right)k_{mbf} - \left( {N - 1} \right)\varphi_{2} \left( {k_{mbf} - k_{s2} } \right)}}} \right)\left( {\frac{{k_{s1} + \left( {N - 1} \right)k_{bf} + \varphi_{1} \left( {k_{bf} - k_{s1} } \right)}}{{k_{s1} + \left( {N - 1} \right)k_{bf} - \left( {N - 1} \right)\varphi_{1} \left( {k_{bf} - k_{s1} } \right)}}} \right) \\ & Pr(\alpha \eta - 2{\varvec{R}}f\left[ \eta \right])\theta ^{\prime}\left[ \eta \right] = 0 \\ \end{aligned}$$

22 $$H_{1} = \left( {\frac{{(1 + 0.1008((\varphi_{1} )^{0.69574} \left( {dp_{1} )^{0.44708} + (\varphi_{2} )^{0.69574} \left( {dp_{2} } \right)^{0.44708} } \right)}}{{\left( {\left( {1 - \left( {\varphi_{1} + \varphi_{2} } \right)} \right) + (\varphi_{1} )\left( {\frac{{\rho_{{s_{1} }} }}{{\rho_{bf} }}} \right) + \left( {\varphi_{2} } \right)\left( {\frac{{\rho_{{s_{2} }} }}{{\rho_{bf} }}} \right)} \right)}}} \right)$$

23 $$H_{2} = \left( {\frac{1}{{\left( {\left( {1 - \varphi_{1} - \varphi_{2} } \right) + \varphi_{1} \left( {\frac{{\rho_{{s_{1} }} }}{{\rho_{bf} }}} \right) + \varphi_{2} \left( {\frac{{\rho_{{s_{2} }} }}{{\rho_{bf} }}} \right)} \right)}}} \right)$$

24 $$H_{3} = \left( {\left( {1 - \left( {\varphi_{1} + \varphi_{2} } \right)} \right) + \left( {\varphi_{1} } \right)\left( {\frac{{\rho_{{cps_{1} }} }}{{\rho_{cpbf} }}} \right) + (\varphi_{2} )\left( {\frac{{\rho_{{cps_{2} }} }}{{\rho_{cpbf} }}} \right)} \right)$$

25 $$D_{1} = \left( {\frac{{k_{s2} + \left( {N - 1} \right)k_{mbf} + \varphi_{2} \left( {k_{mbf} - k_{s2} } \right)}}{{k_{s2} + \left( {N - 1} \right)k_{mbf} - \left( {N - 1} \right)\varphi_{2} \left( {k_{mbf} - k_{s2} } \right)}}} \right)$$

26 $$D_{2} = \left( {\frac{{k_{s1} + \left( {N - 1} \right)k_{bf} + \varphi_{1} \left( {k_{bf} - k_{s1} } \right)}}{{k_{s1} + \left( {N - 1} \right)k_{bf} - \left( {N - 1} \right)\varphi_{1} \left( {k_{bf} - k_{s1} } \right)}}} \right)$$

27 $$\omega = D_{1} D_{2}$$

Putting values of (Eq. 22), (Eq. 23), (Eq. 24), (Eq. 25), (Eq. 26) and (Eq. 27) in Eqs. (Eq. 20) and (Eq. 21) or final result are

28 $$H_{1} f^{\prime\prime\prime\prime}\left[ \eta \right] - \alpha (3f^{\prime\prime}\left[ \eta \right] + \eta f^{\prime\prime\prime}\left[ \eta \right]) - 2{\varvec{R}}f\left[ \eta \right]f^{\prime\prime}\left[ \eta \right] - H_{2} M f^{\prime\prime}[\eta ] = 0$$

29 $$\theta ^{\prime\prime}\left[ \eta \right] + H_{3} \omega Pr(\alpha \eta - 2{\varvec{R}}f\left[ \eta \right])\theta ^{\prime}\left[ \eta \right] = 0$$

#### Solution of the problem

We followed the RK methodology with the inclusion of shooting methods for the solution purpose of the existing flow model. The following replacement remain ingredient to start the process:

$$q_{1}^{*} = f\left[ \eta \right], \quad q_{2}^{*} = f^{\prime}\left[ \eta \right], \quad q_{3}^{*} = f^{\prime\prime}\left[ \eta \right],\quad q_{4}^{*} = f^{\prime\prime\prime}\left[ \eta \right],\quad q_{5}^{*} = \theta \left[ \eta \right],\quad q_{6}^{*} = \theta ^{\prime}\left[ \eta \right]$$

First, transform the model system of ODEs (Eq. 28) and (Eq. 29) in the following pattern,

$$\begin{aligned} & f^{\prime\prime\prime\prime}\left[ \eta \right] = \frac{1}{H}(f^{\prime\prime\prime}\left[ \eta \right]\left( { - \alpha \eta + {\varvec{R}}f\left[ \eta \right]} \right) + f^{\prime\prime}\left[ \eta \right]\left( {3\alpha + 2{\varvec{R}}f^{\prime}\left[ \eta \right]} \right) + H_{2} = Mf_{\eta \eta } ) \\ & \theta^{\prime\prime}\left[ \eta \right] = - H_{3} \omega Pr( - \alpha \eta + 2{\varvec{R}}f\left[ \eta \right])\theta ^{\prime}\left[ \eta \right] \\ \end{aligned}$$

By interchanging, embedded in Eq. (25), the following system is attained:

$$\left[ {\begin{array}{*{20}c} {q_{1}^{*{\prime}} } \\ {q_{2}^{*{\prime}} } \\ {q_{3}^{*{\prime}} } \\ {q_{4}^{*{\prime}} } \\ {q_{5}^{*\prime}} \\ {q_{6}^{*{\prime}} } \\ \end{array} } \right] = \left[ {\begin{array}{*{20}c} {q_{2}^{*} } \\ {q_{3}^{*} }\\ {q_{4}^{*} } \\ {\frac{1}{H1}\left( {f^{\prime\prime\prime}\left[ \eta \right]\left( { - \alpha \eta + Rf\left[ \eta \right]} \right) + f^{\prime\prime}\left[ \eta \right]\left( {3\alpha + 2Rf^{\prime}\left[ \eta \right]} \right) + H_{2} Mf_{\eta \eta } } \right) } \\ {q_{6}^{*} } \\ { - H_{3} \omega Pr\left( { - \alpha \eta + 2Rf\left[ \eta \right]} \right)\theta ^{\prime}\left[ \eta \right]} \\ \end{array} } \right]$$

Consequently, the initial condition are:

$$\left[ {\begin{array}{*{20}c} {q_{{1\left( {{\eta } = { } - 1} \right)}}^{*} } \\ {q_{{2\left( {{\eta } = { } - 1} \right)}}^{*} } \\ {q_{{3\left( {{\eta } = { }1} \right)}}^{*} } \\ {q_{{4\left( {{\eta } = { }1} \right)}}^{*} } \\ {q_{{5\left( {{\eta } = - { }1} \right)}}^{*} } \\ {q_{{6\left( {{\eta } = { }1} \right)}}^{*} } \\ \end{array} } \right] = \left[ {\begin{array}{*{20}c} { - 1} \\ 0 \\ a \\ b \\ 1 \\ 0 \\ \end{array} } \right]$$

where a and b missing initial conditions, now, above system with suitable initial condition (gauss values) is solved until achieved the required accuracy with help of Mathematica.

## Result and discussion:

**Figure 2:**
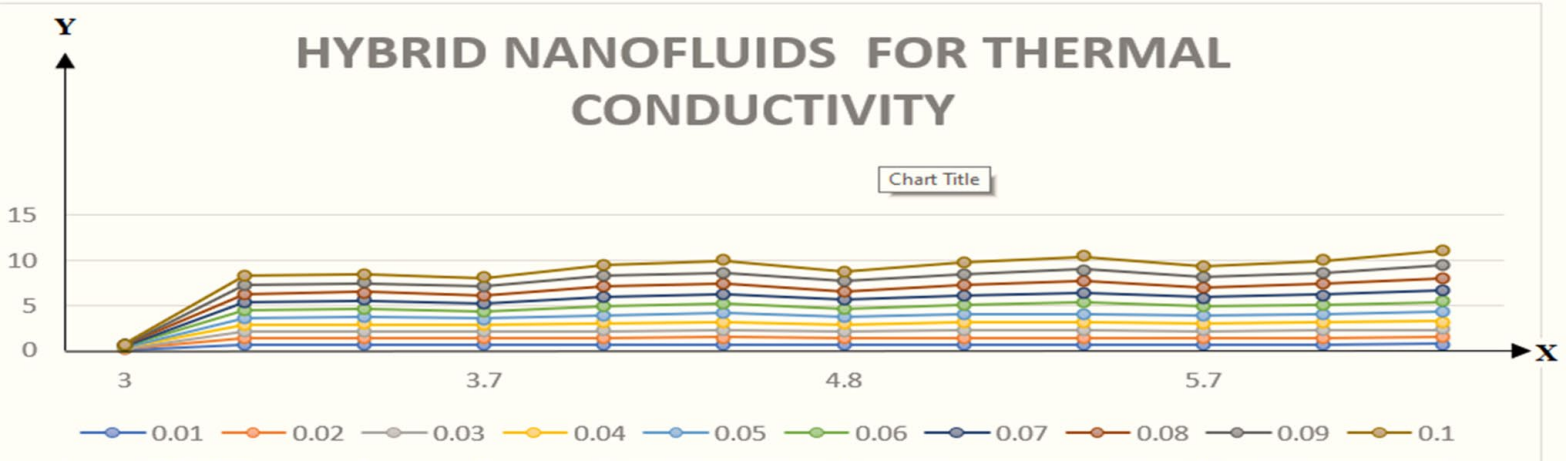
Suggested thermal conductivity behavior for *HN*_*fd*_.

**Figure 3:**
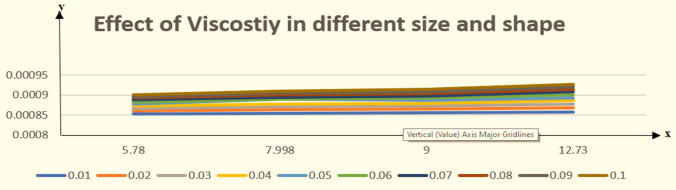
Influence of the scale of hybrid NPs under the viscosity.

**Figure 4:**
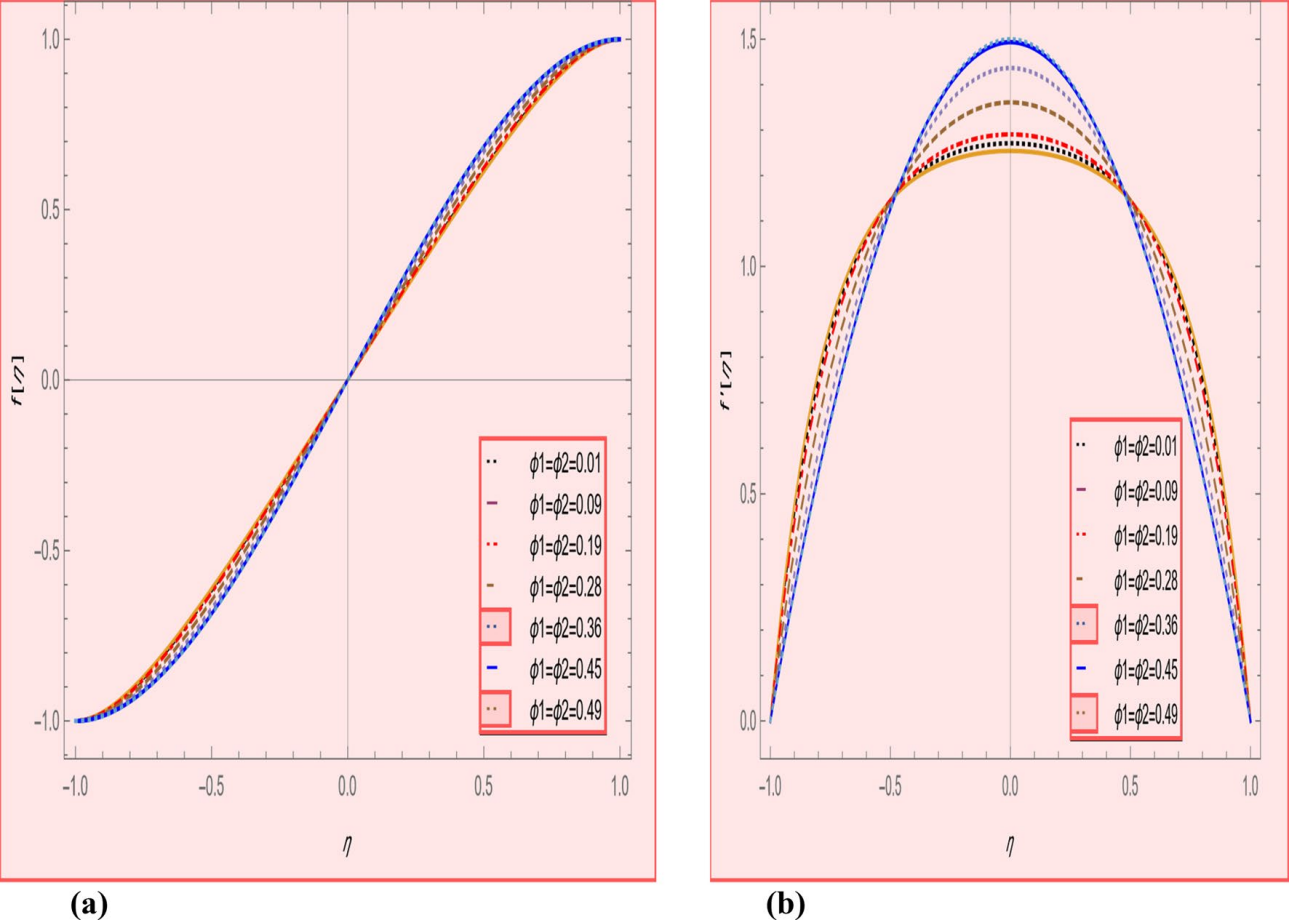
(**a**) Axial, (**b**) radial velocity profile effect in volume fraction for $\alpha = - 4,R = - 1,M = 1,SC = 1,Pr = 6.2.$

**Figure 5:**
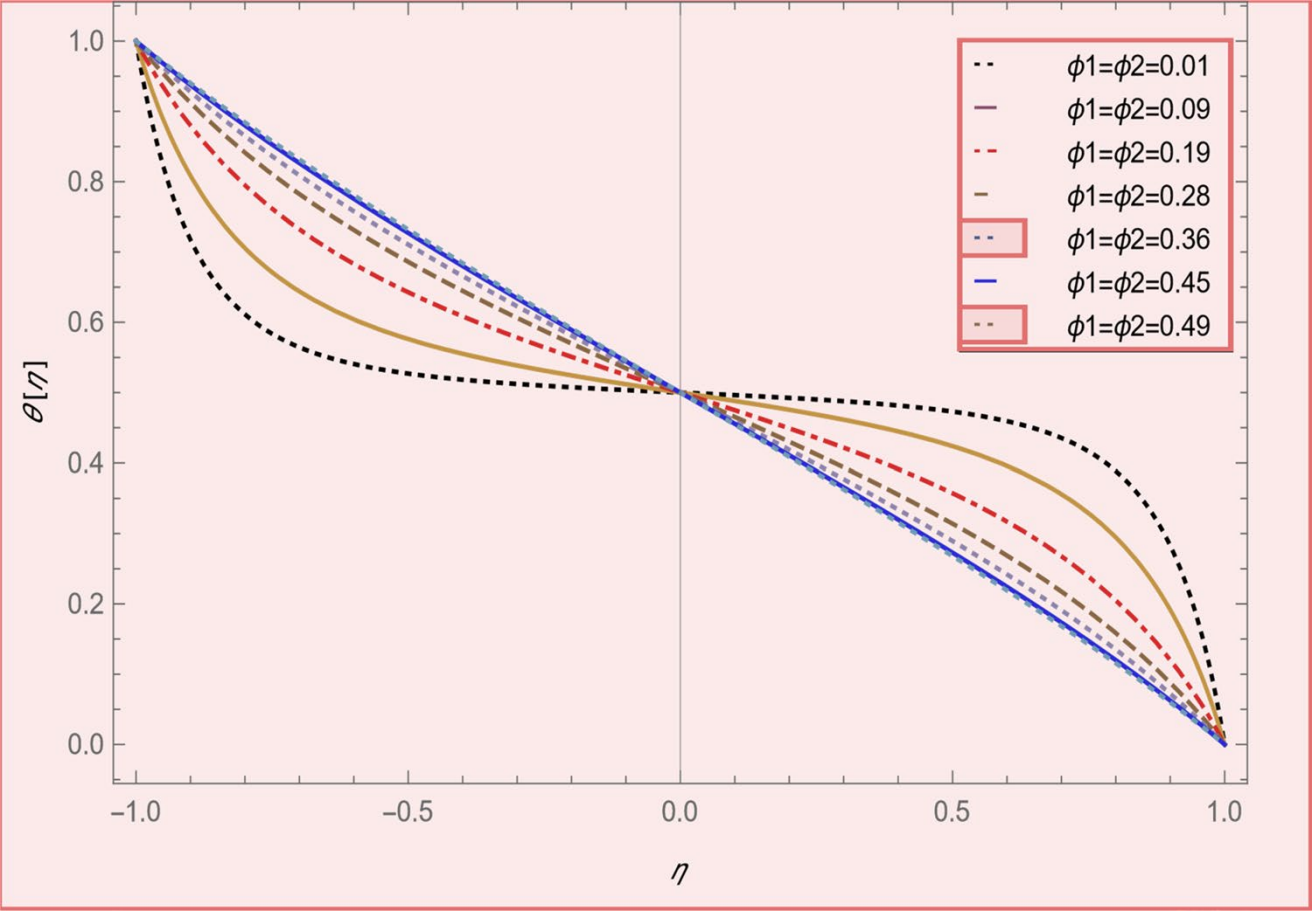
Temperature profile effect in volume fraction for $\alpha = - 4,R = - 1,M = 1,SC = 1,Pr = 6.2$.

**Figure 6:**
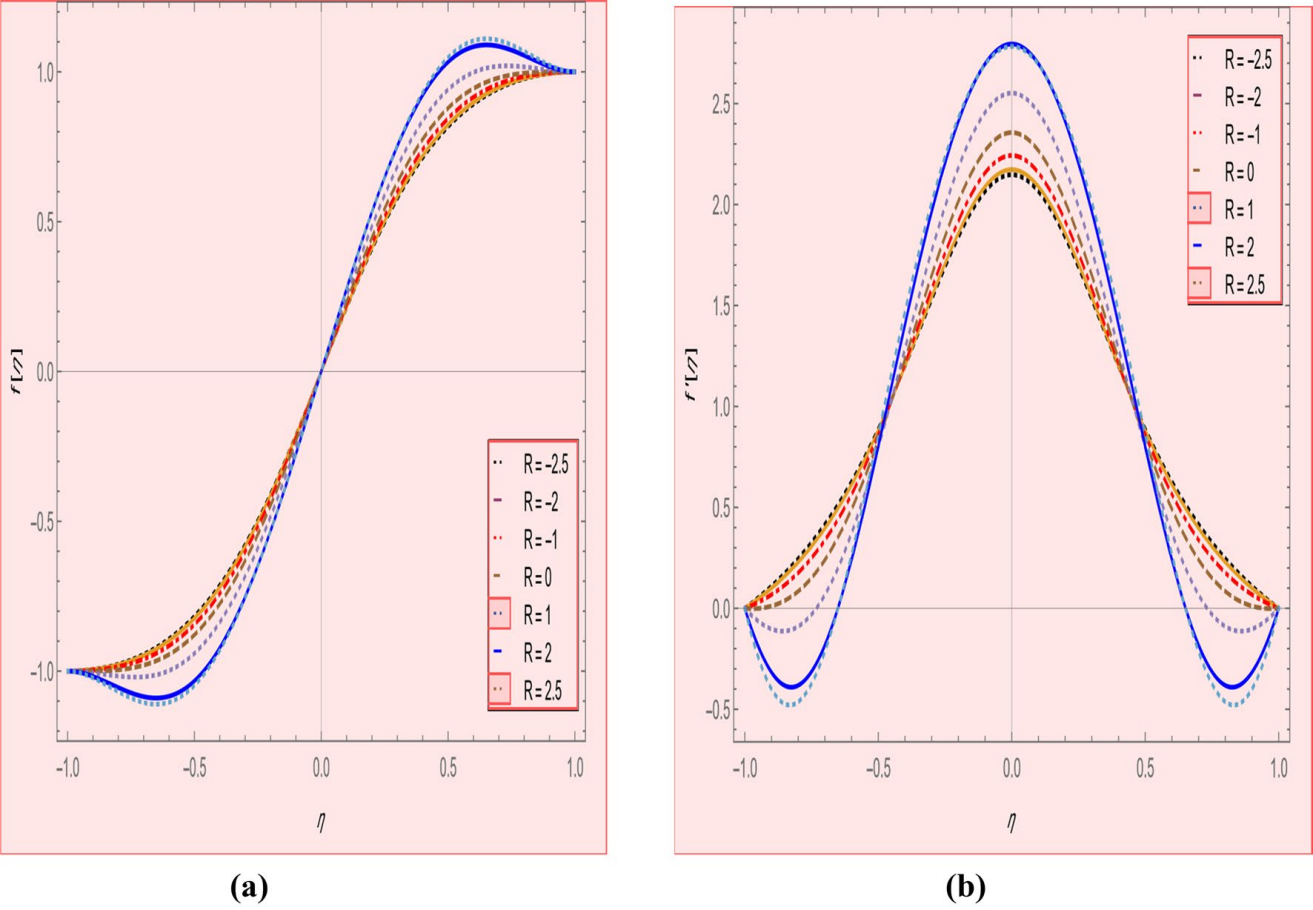
(**a**) Axial, (**b**) redial velocity profile effect in Reynold number for $\alpha = 5,\varphi_{1} = \varphi_{2} = 0.01,M = 1,SC = 1,Pr = 6.2$.

**Figure 7:**
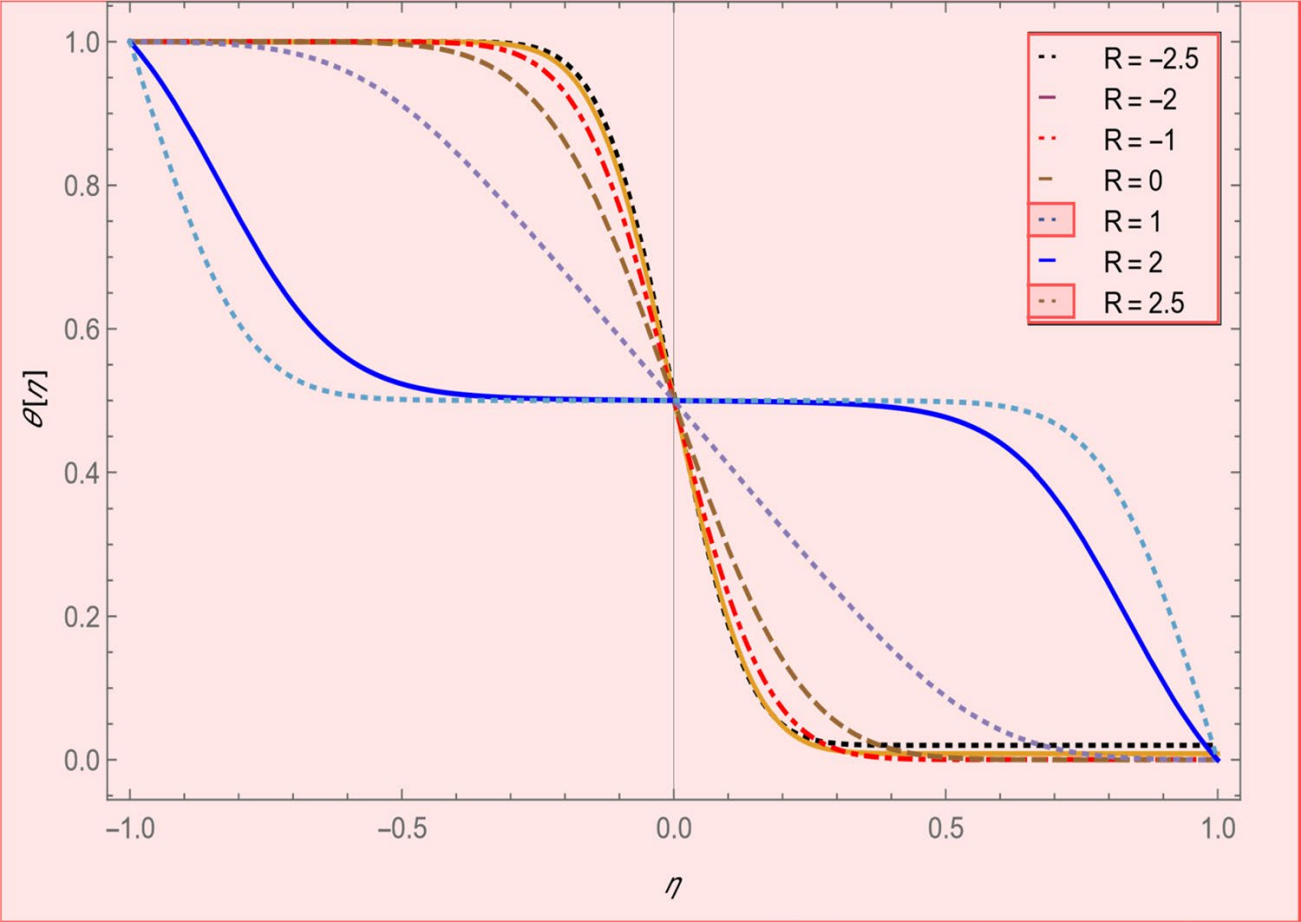
Temperature profile effect permeable Reynold number for $\alpha = 5,\varphi_{1} = \varphi_{2} = 0.01,M = 1,SC = 1,Pr = 6.2.$

**Figure 8:**
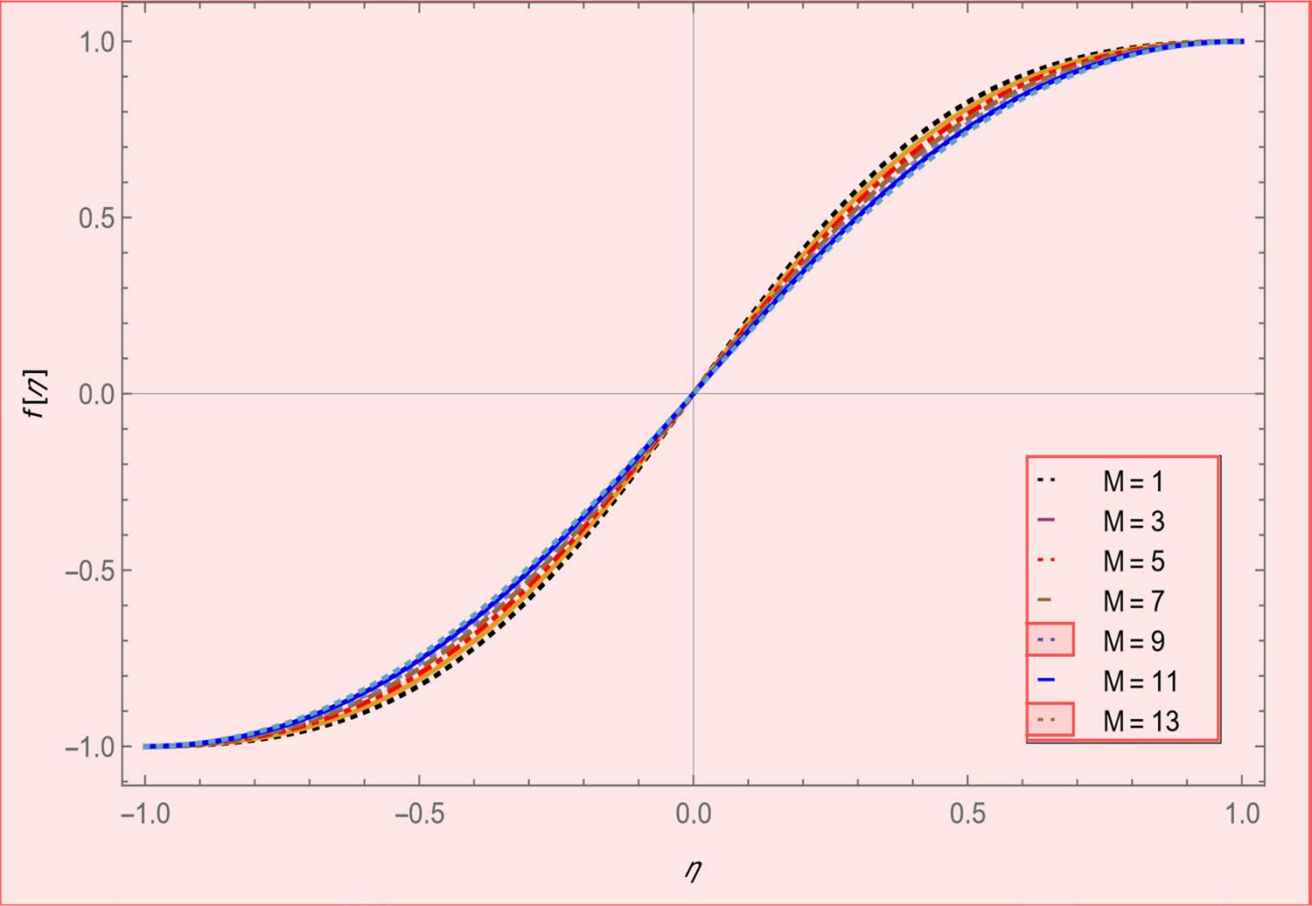
Axial velocity profile effect in magnetic parameter for $\alpha = 4,R = - 1,\varphi_{1} = \varphi_{2} = 0.04,SC = 1,Pr = 6.2.$

**Figure 9:**
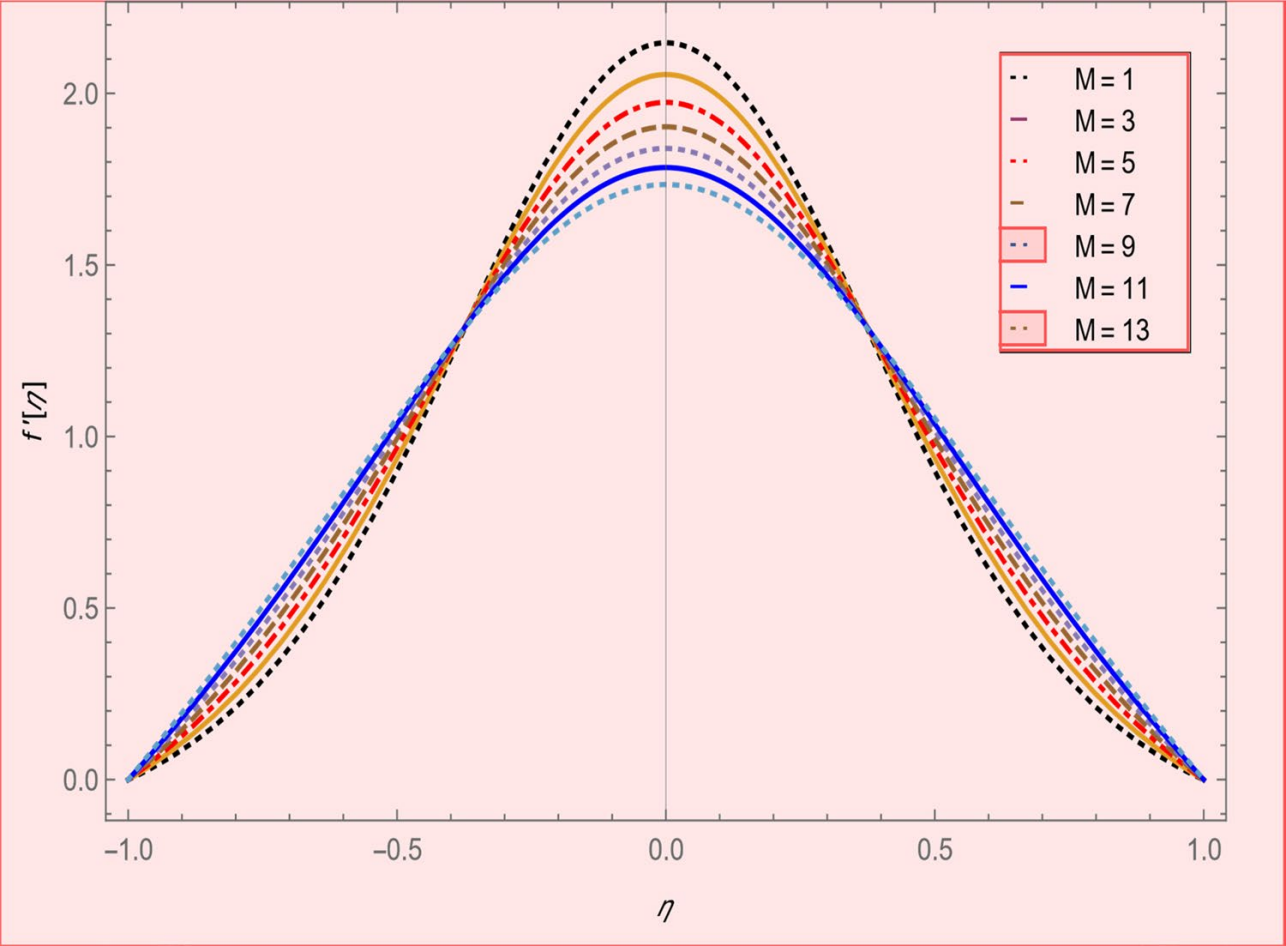
Radial velocity effect in magnetic parameter profile for $\alpha = 4,R = - 1,\varphi_{1} = \varphi_{2} = 0.04,SC = 1,Pr = 6.2$.

**Figure 10:**
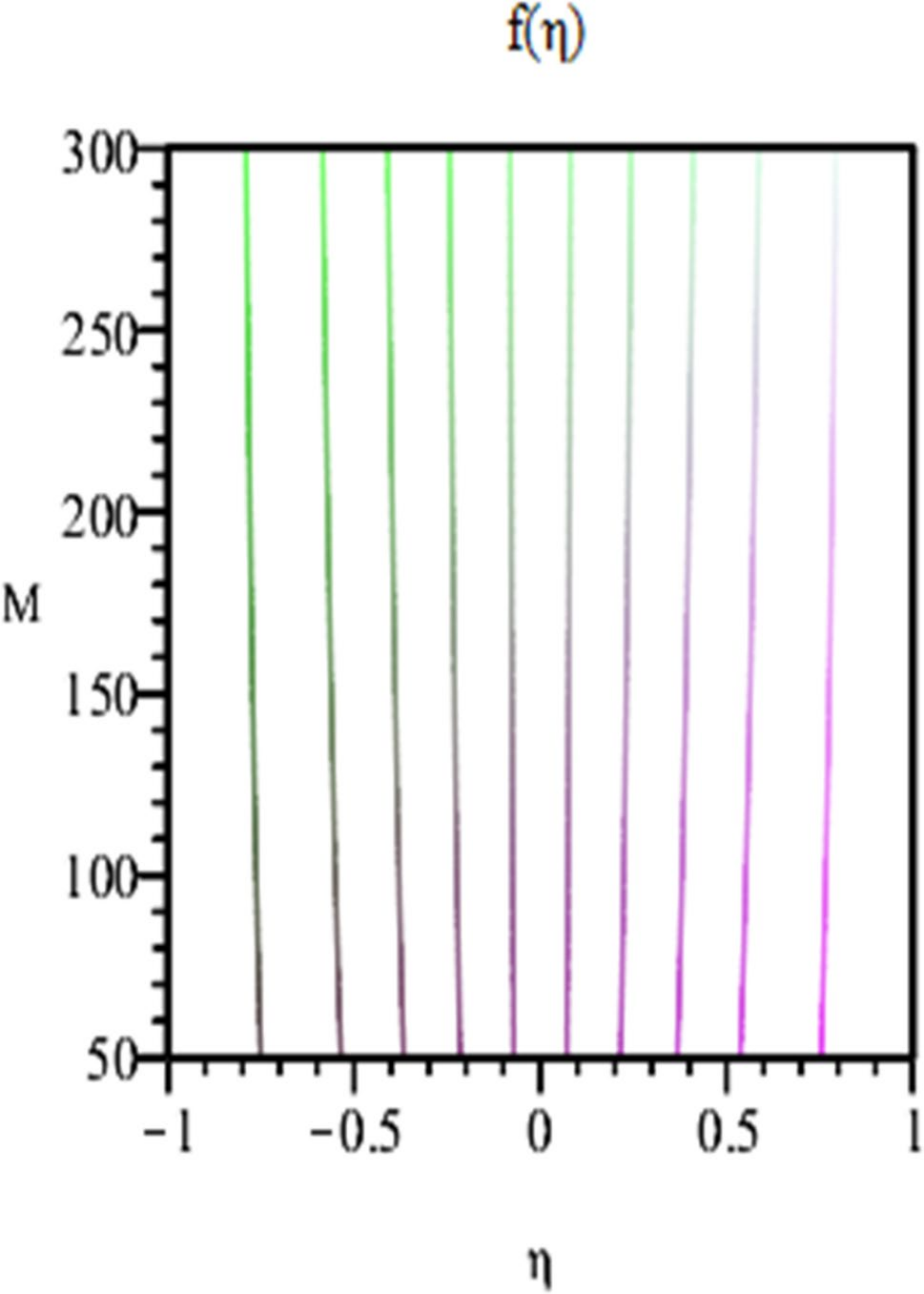
Contour of Axial velocity for $\varphi_{1} = \varphi_{2} = 0.01, Sc = 1,\alpha = 3,Re = - 1, Pr = 6.2$.

**Figure 11:**
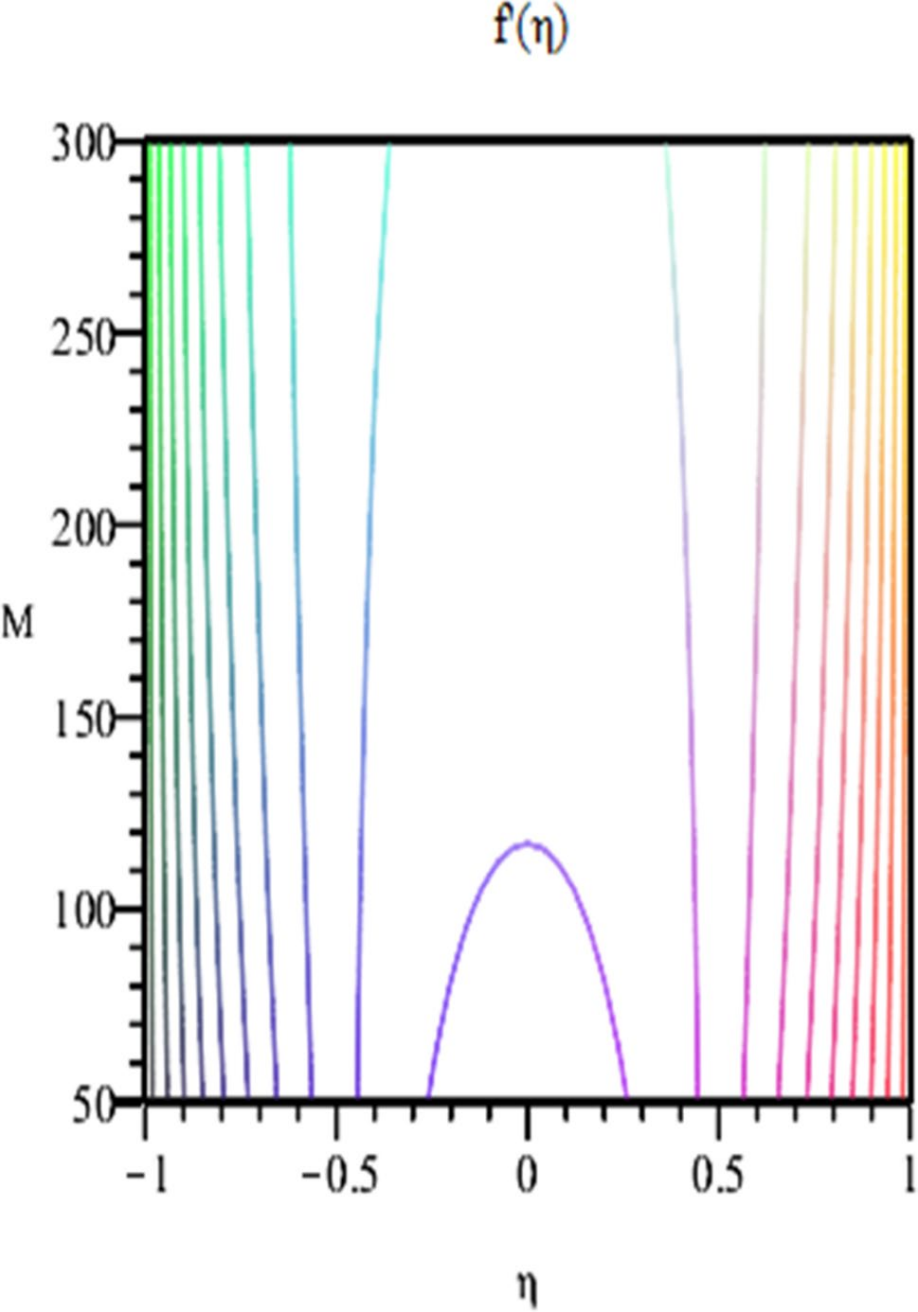
Contour of Radial velocity for $\varphi_{1} = \varphi_{2} = 0.01, Sc = 1,\alpha = 3,Re = - 1, Pr = 6.2$.

**Figure 12:**
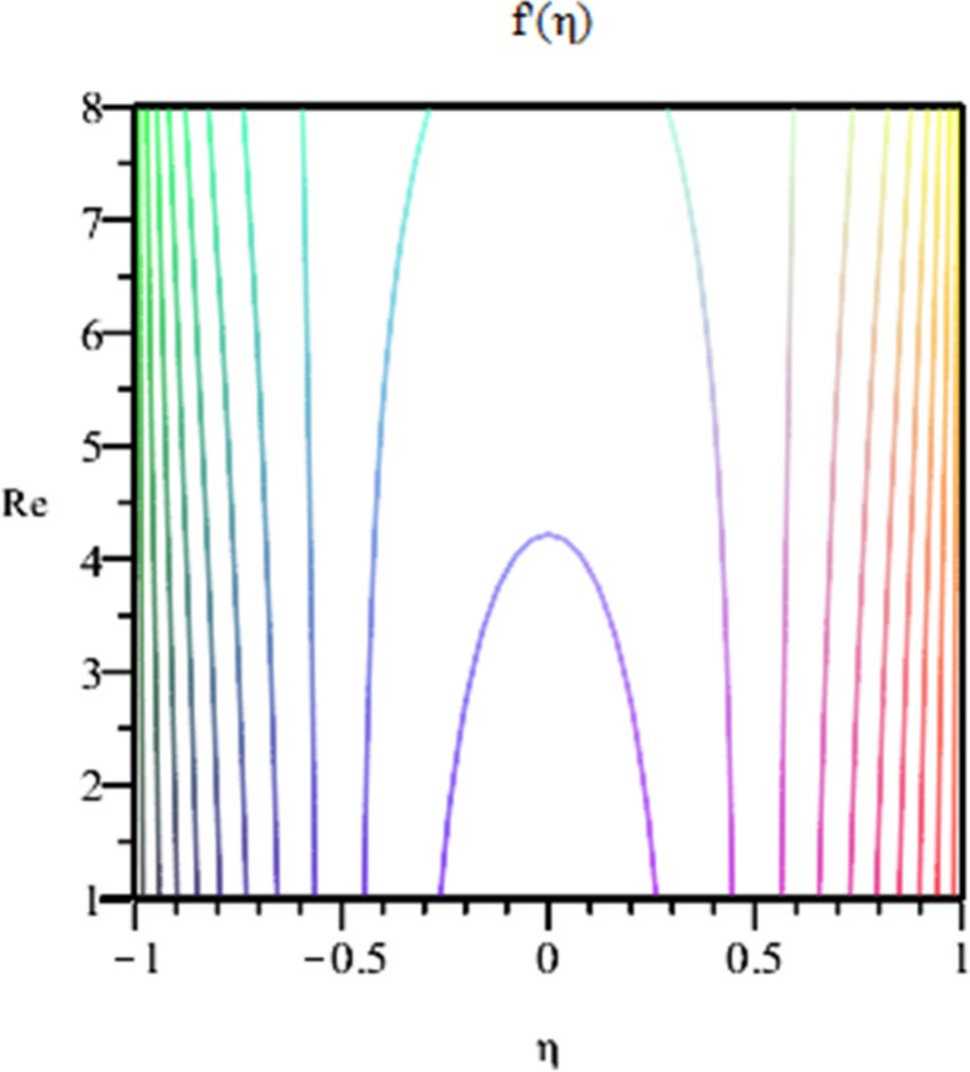
Contour of Radial velocity with increasing of Reynolds number fields for $\varphi_{1} = \varphi_{2} = 0.01, Sc = 1,\alpha = 3,M = 50, Pr = 6.2$.

This section is presented to elaborate the impact of flow concerning equations like expansion/contraction ratio parameter ${\varvec{\alpha}}$, permeable Reynold parameter, magnetic parameter, volume friction parameter on velocity and temperature profile are elaborated through Figs. 2, 3, 4, 5, 6, 7, 8, 9, 10, 11, 12. In addition quantities of engineering interest like shear stress coefficients at lower and upper walls along with heat fluxes are computed numerically against involved variables. Assurance of presently computed data is done by constructing comparison with previously published literature. Thermophysical features like specific heat, density and thermal conductivity of nanoparticles and base fluid is numerically presented in Table 1. Table 2 expresses variation in the effect of shape and size factor of hybrid nanoparticles on heat transfer coefficient by fixing $\varphi_{1} = \varphi_{2} = 0.01, Sc = 1,M = 1,R = - 1, Pr = 6.2,\alpha = - 1$. It is seen from attained values that by increasing magnitudes of ${\varvec{n}}_{1}$ and ${\varvec{n}}_{2}$ the coefficient of convective heat transfer increases. Tables 3 and 4 demonstrates the influence of the $\varphi_{2} ,R, \alpha$ on skin fraction and the Nusselt amount for (Cu–water) and hybridized $({\text{TiO}}_{2} - {\text{Cu}}/water)$ nanofluid for various shapes (sphere, stone, cylindrical, plates) of nanoparticles. It is established that nanofluid and hybrid nano fluid has correlation with skin friction and heat flux coefficients. It is also evident that the amount of Nusselt rises with $\varphi_{2} \; {\text{and}}\; R$ and decreases against α. It is also depicted that skin friction coefficient for hybrid nanofluid is lower than the skin friction coefficient for nanofluid and the skin friction coefficient for hybrid nanofluid is higher than the skin friction coefficient for nanofluid which indicates that hybridized nano fluid is much better that ordinary nanofluid. The effect of Prandtl number and volume fraction $\varphi_{1} \;{\text{and}} \;\varphi_{2}$ on Nusselt number and skin friction coefficient are shown in Table 5. Table 5 also shows that by increasing volume fractions $\varphi_{1} \;{\text{and}} \;\varphi_{2}$ and by fixing Prandtl number the skin friction and Nusselt number increases. The reason behind this fact is that Prandtl number is the ratio of diffusive momentum to thermal diffusivity so by increasing (Pr) momentum diffusivity enhances which as an outcome raises heat flux coefficient. Table 6 shows impact of magnetic parameter and permeable Reynold number on skin friction and heat transfer in Table 6. It is observed that for R < 0 or R > 0 and $\alpha$ < 0 or $\alpha$ > 0 the skin friction coefficient and Nusselt number increases against (M). Table 7 displays the effect of injection/suction parameter $\alpha$ skin friction coefficient and Nusselt number rises. It is also evidenced that $R = 1$ and $R = - 1$ and by assuming $(\alpha )$ less or greater than zero skin friction coefficient and heat transfer of Nusselt number decrease for both porous disks. Table 8 shows impact of different physical parameters which effect skin friction and Nusselt number coefficients. It is found that by decreasing magnetic parameter skin friction coefficient decreases and the Nusselt number and associated thermal boundary layer thickness increases. Specifically it is noticed that by increasing (Pr) skin friction coefficient decreases and Nusselt number increases. The reason behind this fact is that by increasing Prandtl number momentum diffusivity enhances which as an outcome increases average kinetic energy of molecules and heat flux increases and resistance provided by surface to fluid decreases. Table 9 shows credibility of work by constructing comparison with previously published literature presented by Kashif et al.^46^. Table 10 shows numerical stability of results for f (−1), f' (−1) and f'' (−1) at different values of η.

**Table 1:** Thermophysical features of *HN*_*fd*_ and NP’s.

Physical properties	${\text{H}}_{2}$ O (f)	Fe_3_O_4_	Al_2_O_3_	${\text{TiO}}_{2}$	Cu
$\rho$ (kg m^−3^)	997.0	5180	3970	4250	8933
(J k g^−1^ k^−1^	4180	670	765	686.2	385
$\kappa \;\left( {{\text{wm}}^{ - 1} \;{\text{k}}^{ - 1} } \right)$	0.6071	9.7	40	8.9538	400

**Table 2:** Effect of shape and size factor of *HN*_*fd*_ on heat transfer coefficient by fixing $\varphi_{1} = \varphi_{2} = 0.01, Sc = 1,M = 1,R = - 1, Pr = 6.2,\alpha = - 1$.

Types	$n_{1} = 3$				$n_{2} = 3$			
	$n_{2} = 3$	$n_{2} = 3.7$	$n_{2} = 4.8$	$n_{2} = 5.7$	$n_{1} = 3$	$n_{1} = 3.7$	$n_{1} = 4.8$	$n_{1} = 5.7$
HNF1	$0.01755$	$0.01798$	$0.01866$	$0.01922$	$0.01755$	$0.01785$	$0.01829$	$0.01862$
HNF2	$0.01763$	$0.01791$	$0.01832$	$0.01863$	$0.01763$	$0.01808$	$0.01881$	$0.01941$
HNF3	$0.01789$	$0.01835$	$0.01909$	$0.01969$	$0.01789$	$0.01831$	$0.01897$	$0.01949$
HNF4	$0.01814$	$0.0186$	$0.01933$	$0.01994$	$0.01814$	$0.0186$	$0.01934$	$0.01994$

**Table 3:** Numerical variation in shear stress at the lower porous wall with different shape factors for $\varphi_{2} ,\varphi_{1} = 0,R$, $\alpha$ ($Cu -$water) (*N*_*fd*_).

Cu–water							
$\varphi_{2}$	$R$	$\alpha$	$\left| { C_{f} } \right|_{\eta = - 1}$	$\left| { Nu_{z} \left( 3 \right)} \right|_{\eta = - 1}$	$\left| { Nu_{z} \left( {3.7} \right)} \right|_{\eta = - 1}$	$\left| { Nu_{z} \left( {4.8} \right)} \right|_{\eta = - 1}$	$\left| { Nu_{z} \left( {5.7} \right)} \right|_{\eta = - 1}$
0.02	0.1	1	2.4499	0.09544	0.09690	0.09894	0.10042
0.03			2.4416	0.10011	0.10229	0.10533	0.10753
0.04			2.4341	0.10482	0.10772	0.11175	0.11465
0.05			2.4275	0.10958	0.11318	0.11818	0.12177
0.05	0	1	2.3933	0.063041	0.06592	0.06998	0.7294
	0.1		$\user2{ }2.4275$	$0.10958$	$0.11318$	$0.11818$	$0.12176$
	0.2		$2.4643$	$0.18506$	$0.18893$	$0.19424$	$0.19800$
	0.3		$2.5036$	$0.30192$	$0.30502$	$0.30921$	$0.31215$
0.05	0.1	0.3	$2.9831$	$0.44841$	$0.44938$	$0.45068$	$0.45158$
		0.5	$2.8218$	$0.30755$	$0.31061$	$0.31476$	$0.31766$
		0.7	$2.6626$	$0.20636$	$0.21021$	$0.21545$	$0.21916$
		1	$2.4275$	$0.10958$	$0.11318$	$0.11818$	$0.12177$

**Table 4:** Numerical variation in shear stress against $\varphi = \varphi_{1} = \varphi_{2} , R$ ,$\alpha { }({\text{TiO}}_{2} - {\text{Cu/water)}}$ (*HN*_*fd*_).

$${\text{TiO}}_{{2}} {\text{ - Cu/water}}$$							
$\varphi$	$R$	$\alpha$	$\left| { C_{f} } \right|_{\eta = - 1}$	$\left| { Nu_{z} \left( 3 \right)} \right|_{\eta = - 1}$	$\left| { Nu_{z} \left( {3.7} \right)} \right|_{\eta = - 1}$	$\left| { Nu_{z} \left( {4.8} \right)} \right|_{\eta = - 1}$	$\left| { Nu_{z} \left( {5.7} \right)} \right|_{\eta = - 1}$
0.02	0.1	1	$2.3728$	$0.10655$	$0.11044$	$0.11629$	$0.12087$
0.03			$2.3345$	$0.11709$	$0.12298$	$0.13178$	$0.13862$
0.04			$2.3023$	$0.12782$	$0.13569$	$0.14737$	$0.15635$
0.05			$2.2761$	$0.13869$	$0.14849$	$0.16290$	$0.17385$
0.05	0	1	$2.2412$	$0.08707$	$0.09569$	$0.10876$	$0.11899$
	0.1		$2.2761$	$0.13869$	$0.14849$	$0.16290$	$0.17385$
	0.2		$\user2{ }2.3141$	$0.21577$	$0.22543$	$0.23926$	$0.24950$
	0.3		$2.3557$	$0.32667$	$0.32667$	$0.34377$	$0.35099$
0.05	0.1	0.3	$2.9276$	$0.45573$	$0.45784$	$0.46074$	$0.46281$
		0.5	$2.7380$	$0.33074$	$0.33780$	$0.34765$	$0.35478$
		0.7	$2.5510$	$0.23612$	$0.24554$	$0.25893$	$0.26880$
		1	$2.2760$	$0.13869$	$0.14849$	$\user2{ }0.16290$	$0.17386$

**Table 5:** Variation in ${C}_{f}$ and ${Nu}_{z}$ against ($Pr$) and volume fraction ${\varphi }_{1},{\varphi }_{2}$ for (Cu–${TiO}_{2}/$water) hybrid nanofluid when $\alpha =-1,R=-1,M=1.$

$\varphi_{1} = \varphi_{2}$	$Pr$	$\left| {C_{f} } \right|_{\eta = - 1}$	$\left| {Nu_{z} } \right|_{\eta = - 1}$
0.01 = 1%	5.5	$3.3953$	$0.0269$
0.02 = 2%	5.5	$3.3959$	$0.0324$
0.03 = 3%	5.5	$3.3964$	$0.0385$
0.04 = 4%	5.5	$3.3969$	$0.0452$
0.01 = 1%	6.2	$3.3953$	$0.0147$
0.01 = 1%	6.7	$3.3953$	$0.0131$
0.01 = 1%	6.9	$3.3953$	$0.0115$

**Table 6:** Variation in $C_{f}$ and $Nu_{z}$ against (M) and (*R*) for (*HN*_*fd*_) (Cu–${\text{TiO}}_{2} /$water) when $\varphi_{1} = \varphi_{2} = 0.01,\alpha = - 1,Sc = 1,Pr = 6.2.$

*M*	*R*	$\alpha = - 1$		$\alpha = 1$	
		$\left| {C_{f} } \right|_{\eta = - 1}$	$\left| {Nu_{z} } \right|_{\eta = - 1}$	$\left| {C_{f} } \right|_{\eta = - 1}$	$\left| {Nu_{z} } \right|_{\eta = - 1}$
1	− 1.5	$3.1641$	$0.000611$	$2.0856$	$0.0000014$
3	− 1	$3.6742$	$0.018523$	$2.4389$	$0.0000594$
6	− 0.5	$4.3941$	$0.35553$	$3.0194$	$0.0021022$
9	0	$5.1767$	$2.31074$	$3.6834$	$0.0526765$
11	0.5	$5.8919$	$5.4094$7	$4.284$	$0.6776833$
13	1	$6.6616$	$8.46962$	$5.4229$	$5.8166543$
15	1.5	$6.40676$	$6.61847$	$6.2224$	$8.744112$

**Table 7:** Variation in $C_{f}$ and $Nu_{z}$ against $\alpha$ for (*HN*_*fd*_) (Cu–$TiO_{2} /$water) when $\varphi_{1} = \varphi_{2} = 0.01$, $M = 1,Sc = 1,Pr = 6.2.$

$\alpha$	$Re = 1$		$Re = - 1$	
	$\left| {C_{f} } \right|_{\eta = - 1}$	$\left| {Nu_{z} } \right|_{\eta = - 1}$	$\left| {C_{f} } \right|_{\eta = - 1}$	$\left| {Nu_{z} } \right|_{\eta = - 1}$
− 3	$7.1043$	$14.253$	$4.8889$	$1.65409$
− 2	$6.0398$	$11.364$	$$\begin{array}{*{20}c} {4.1151} \\ \end{array}$$	$0.23546$
− 1	$4.9781$	$8.4941$	$3.3953$	$0.017631$
0	$3.9204$	$5.6703$	$2.7374$	$0.0010334$
1	$2.8672$	$3.0214$	$2.1478$	$0.000055$
2	$1.8173$	$1.039$	$1.6314$	$0.0000027$
3	$0.7643$	$0.20338$	$1.1904$	$1.320 \times 10^{ - 7}$

**Table 8:** Variation in skin friction coefficient and Nusselt number at lower wall.

$\alpha$	$R$	$M$	${\varphi }_{1}$	${\varphi }_{2}$	${n}_{1}$	${n}_{2}$	*Pr*	${\left| {C}_{f}\right|}_{\eta =-1}$	${\left| {Nu}_{z}\right|}_{\eta =-1}$
0.1	0.01	1	0.01	0.01	3	3	6.2	3.1080	0.43022
0.2								3.0262	0.35087
0.3								2.9437	0.28423
0.4								2.8623	0.22881
	− 0.02							3.0801	0.33628
	− 0.03							3.0591	0.27383
	− 0.04							3.0485	0.24645
		1						2.7231	0.43041
		2						2.5132	0.43053
		3						2.2893	0.43065
			0.02					3.0999	0.43221
			0.03					3.0922	0.43419
			0.04					3.0848	0.43607
				0.02				3.1031	0.43198
				0.03				3.0984	0.43367
				0.04				3.0939	0.43533
					3.7			3.1080	0.43022
					4.8			3.1080	0.43066
					5.7			3.1080	0.43118
						3.7		3.1080	0.43051
						4.8		3.1080	0.43089
						5.7		3.1080	0.43118
							4.5	3.1080	0.44852
							5.2	3.1080	0.44094
							5.5	3.1080	0.43767

**Table 9:** Comparison of Nusselt number at lower porous wall with results published by Kashif et al.^46^ against $\varphi$ and $\alpha .$

$\varphi$^50^	$\varphi 1 + \varphi 2$	$\alpha > 0$^46^	Present work	$\alpha < 0$^46^	Present work
0%	0%	1.6794	1.6797	3.1664	3.1766
5%	5%	1.9174	1.9197	3.6112	3.6123
10%	10%	2.2135	2.2158	4.1606	4.1616
15%	15%	2.5839	2.5849	4.8430	4.8550
20%	20%	3.0519	3.0529	5.6988	5.6998

**Table 10:** Data for the Numerical Stability.

$\eta$	$f\left( { - 1} \right)$	$f^{\prime}\left( { - 1} \right)$	$f^{\prime\prime}\left( { - 1} \right)$
− 1	− 1	0	$$\begin{array}{*{20}c} {2.8682185248890812} \\ \end{array}$$
$- 0.9$	$- 0.986004632148899$	$$\begin{array}{*{20}c} {0.2762900573162856} \\ \end{array}$$	$$\begin{array}{*{20}c} {2.6513635499005477} \\ \end{array}$$
$- 0.8$	$$\begin{array}{*{20}c} { - 0.9455224728264963} \\ \end{array}$$	$$\begin{array}{*{20}c} {0.5291984277529419} \\ \end{array}$$	$$\begin{array}{*{20}c} {2.4022339523123413} \\ \end{array}$$
$- 0.7$	$$\begin{array}{*{20}c} { - 0.8810372014384086} \\ \end{array}$$	$$\begin{array}{*{20}c} {0.7559667541088714} \\ \end{array}$$	$$\begin{array}{*{20}c} {2.129942115638948} \\ \end{array}$$
$- 0.6$	$$\begin{array}{*{20}c} { - 0.7952655915371383} \\ \end{array}$$	$$\begin{array}{*{20}c} {0.9546625435491819} \\ \end{array}$$	$$\begin{array}{*{20}c} {1.8418610445096326} \\ \end{array}$$
$- 0.5$	$$\begin{array}{*{20}c} { - 0.6910837969233515} \\ \end{array}$$	$$\begin{array}{*{20}c} {1.1240010434185939} \\ \end{array}$$	$$\begin{array}{*{20}c} {1.5435889317798919} \\ \end{array}$$
$- 0.4$	$$\begin{array}{*{20}c} { - 0.5714712122097215} \\ \end{array}$$	$$\begin{array}{*{20}c} {1.263175625521357} \\ \end{array}$$	$$\begin{array}{*{20}c} {1.2391316972955884} \\ \end{array}$$
$- 0.3$	$$\begin{array}{*{20}c} { - 0.4394701755700228} \\ \end{array}$$	$$\begin{array}{*{20}c} {1.371712201118385} \\ \end{array}$$	$$\begin{array}{*{20}c} {0.93118511715582} \\ \end{array}$$
$- 0.2$	$$\begin{array}{*{20}c} { - 0.29815875720869417} \\ \end{array}$$	$1.4493534762608988$	$$\begin{array}{*{20}c} {0.6214396059796121} \\ \end{array}$$
$- 0.1$	$$\begin{array}{*{20}c} { - 0.1506336107568336} \\ \end{array}$$	$$\begin{array}{*{20}c} {1.4959731677879262} \\ \end{array}$$	$$\begin{array}{*{20}c} {0.31087067312291067} \\ \end{array}$$
$0$	$$\begin{array}{*{20}c} {1.55484340680353 \times 10^{ - 10} } \\ \end{array}$$	$$\begin{array}{*{20}c} {1.5115177949361303} \\ \end{array}$$	$$\begin{array}{*{20}c} {1.2913497632327 \times 10^{ - 9} } \\ \end{array}$$
0.1	$$\begin{array}{*{20}c} {0.1506336107568336} \\ \end{array}$$	$$\begin{array}{*{20}c} {1.4959731683236508} \\ \end{array}$$	$$\begin{array}{*{20}c} {0.31087067312291067} \\ \end{array}$$
0.2	$$\begin{array}{*{20}c} {0.29815875720869417} \\ \end{array}$$	$1.4493534762608988$	$$\begin{array}{*{20}c} {0.6214396059796121} \\ \end{array}$$
0.3	$$\begin{array}{*{20}c} {0.4394701755700228} \\ \end{array}$$	$$\begin{array}{*{20}c} {1.371712201118385} \\ \end{array}$$	$$\begin{array}{*{20}c} {0.93118511715582} \\ \end{array}$$
0.4	$$\begin{array}{*{20}c} {0.5714712122097215} \\ \end{array}$$	$$\begin{array}{*{20}c} {1.263175625521357} \\ \end{array}$$	$$\begin{array}{*{20}c} {1.2391316972955884} \\ \end{array}$$
.5	$$\begin{array}{*{20}c} {0.6910837969233515} \\ \end{array}$$	$$\begin{array}{*{20}c} {1.1240010434185939} \\ \end{array}$$	$$\begin{array}{*{20}c} {1.5435889317798919} \\ \end{array}$$
0.6	$0.7952655915371383$	$$\begin{array}{*{20}c} {0.9546625435491819} \\ \end{array}$$	$$\begin{array}{*{20}c} {1.8418610445096326} \\ \end{array}$$
0.7	$0.8810372014384086$	$0.7559667541088714$	$$\begin{array}{*{20}c} {2.129942115638948} \\ \end{array}$$
0.8	$$\begin{array}{*{20}c} {0.9455224728264963} \\ \end{array}$$	$$\begin{array}{*{20}c} {0.5291984277529419} \\ \end{array}$$	$$\begin{array}{*{20}c} {2.4022339523123413} \\ \end{array}$$
0.9	$0.986004632148899$	$$\begin{array}{*{20}c} {0.5291984277529419} \\ \end{array}$$	$$\begin{array}{*{20}c} {2.6513635499005477} \\ \end{array}$$
1	1	0	$$\begin{array}{*{20}c} {2.8682185248890812} \\ \end{array}$$

Figure 2 expresses variation in thermal conductivity against $\varphi_{1}$ and $\varphi_{2}$ and by considering four different shapes (spherical, plates, bricks, cylindrical) of hybrid nanoparticles. We observed that higher thermal conductivity performance is achieved at 5.7 when particles volume fractions is at magnitude 0.1. It means if we gradually increase the numerical values of $\varphi_{1}$ and $\varphi_{2}$ and shape factor along with volume fractions thermal conductivity increases. In Fig. 3 it is observed that the viscosity base fluid (water) depends on volume fraction $\varphi$, diameter ($dp_{1} \;{\text{and}}\; dp_{2}$) of particles. It is manifested that as we increase volume fraction of nanoparticles it causes internal shear stress to enhance which will, in turn, increases the viscosity of (HNFDs). The increase in hydrodynamic diameter of nano structures as a result adsorption and clustering will lead to enhance the viscosity. Furthermore, the effect of viscosity is very high for size factor 12.73 when particles volume fraction $\varphi_{1}$ and $\varphi_{2}$ is 0.1 We observed that in Figs. 2 and 3 if we are increasing the values of volume fraction and shape or size factor increases thermal conductivity and viscosity will arise. Figures 4 and 5 represents the impact of volume fraction parameter on axial, radial velocity components and on temperature profile for fixed values for $\alpha = - 4,R = - 1,M = 1,Sc = 1,Pr = 6.2.$ It is observed that volume fraction parameter causes decrease in axial and radial component of velocity. Physically, it is due to the reason that heat is dissipated with deformation in shape of nanoparticles. In addition uplift in nanoparticle volume fraction will consumes more energy and thermal boundary layer thickness raises. Figures 6, 7 shows effect of permeable Reynold number on the axial and radial velocity and temperature profile. Physically, it is worth noting that the amount of Reynold produces inertial effect and as an outcome viscosity reduces and velocity increases. Figures 8, 9 displays impact of magnetic parameter on axial and radial velocity profile. It is observed that by increasing (M) axial component of velocity increases whereas radial velocity component decreases. This is because of the fact that by increasing (M) Lorentz forces are generated which reduces momentum of fluid particles in axial direction. From this statement, we can conclude that the velocity of fluid is normalized by the transverse application magnetic field. The vibration of the particles within the fluid which is controlled by the Lorentz force is due to the magnetic effect. Figure 10 represents variation in velocity against (M) through contour lines. It is depicted that contour lines are roughly flat in the area of the midpoint of $\eta$ and negligible reducing pattern along the problem's boundary. Figure 11 displays the contour of the influence of variable M on the radial velocity. In Fig. 11 contour lines representing variation in radial component of velocity is sketched which shows optimized change in velocity against $\eta$ at boundaries whereas at center these lines shows zero change. Figure 12 indicates that the contours lines are very similar to the problem's boundaries as opposed to the center of an area and contributes to the radial velocity maximum being placed in the center of an area. The contour of radial velocity with the variance of parameter *R* was seen in Fig. 12. It is also noticed that contour lines expand against the boundaries of the main problem and expand in the major component of the field with an appreciation in the value of R which contributes to a reduction in the radial velocity with a fall in the amount of *R*.

## Conclusion

In this study numerical analysis of MHD hybrid nanofluids flows through porous surfaces is adumbrated. Thermophysical features of metallic and ceramic-metallic nanocomposites are combined with hybrid nanofluid. Evaluation about shape, size factors and particle volume fraction on velocity, temperature profile is executed. Numerical and graphical consequences are gained regarding skin friction coefficient and Nusselt number.

Some key findings are itemized as below

1. Nusselt number at* n* = 5.7 shows good results in MMNC than shape factors at both porous walls.
2. Magnetic parameters have a significant effect on skin friction and Nusselt number for hybrid flow.
3. In absence of injection/suction all governing parameters at lower porous surface show good performance as compared to upper surface.
4. Hybrid nanofluids under the effect of M and *R* indicate better results as compared to nanofluids.
5. For the contracting case Nusselt and skin friction coefficients for hybrid nanofluid show significant results.
6. Increase in volume fraction thermal boundary layer thickness increases but permeable Reynold number reduces thermal boundary layer thickness.

## Abbreviations

$B_{o}$: Uniform magnetic field (T)
$C_{f}$: The total skin friction coefficient
$C_{p}$: Specific heat at constant pressure
$k$: Time depended on coefficient
M: Magnetic parameter
$$P_{r}$$: Prandtl number
r z: Cylindrical coordinates system
*R*: Reynolds number
W: Mass or velocity component along z-axis
Nu: Nusselt number
$$\rho_{hnf}$$: Density for ($$HN_{fd}$$)
$\rho_{s2}$: Density for second solid NP’s
$k_{s1}$: Thermal conductivity for first solid fraction
$k_{mbf}$: Thermal conductivity for shape base fluid
$k_{bf}$: Thermal conductivity for base fluid
${ }\mu_{bf}$: Viscosity based fluid
$\upsilon_{hnf}$: Kinematics viscosity for ($HN_{fd}$)
$F_{\eta }$: The dimensionless radial velocity profile
$\theta_{\eta }$: Dimensionless temperature profile
$\sigma$: Electrical conductivity ((${\text{m}}^{3} \;{\text{A}}^{2}$)/kg)
$$\upsilon$$: Kinematic viscosity ($${\text{m}}^{2}$$/s)
$\mu$: Dynamic viscosity (Pa s)
$$\rho$$: Density (kg/m^3^)
$\rho C_{P}$: Volumetric heat capacity (J/(m^3^ K))
T ($$HN_{fd}$$): Temperature (K)
$A_{1}$: Dimensionless parameter coefficient
$(\rho c_{p} )_{hnf}$: Specific heat capacity for ($HN_{fd}$)
$\rho_{s1}$: Density for first solid NP’s
$k_{hnf}$: Thermal conductivity for ($HN_{fd}$)
$k_{s2}$: Thermal conductivity for second solid fraction
P: Pressure
${ }\mu_{eff}$: Viscosity for effect
dp: Dimeter of particles
$$Nu_{z}$$: Nusselt number

## Subscripts

($$B_{fd}$$): Base fluid
($$HN_{fd}$$): Hybrid Nanofluid
($$N_{fd}$$): Nanofluid
HNF1: $$({\text{Fe}}_{3} {\text{O}}_{4} - {\text{TiO}}_{2} /{\text{H}}_{2} {\text{O}})$$
HNF2: $$({\text{Fe}}_{3} {\text{O}}_{4} - {\text{Al}}_{2} {\text{O}}_{3} /{\text{H}}_{2} {\text{O}})$$
HNF3: $$({\text{Al}}_{2} {\text{O}}_{3} - {\text{TiO}}_{2} /{\text{H}}_{2} {\text{O}})$$/)
HNF4: $$({\text{TiO}}_{2} - {\text{Cu}}/{\text{H}}_{2} {\text{O}})$$
1: First nanoparticle $$\left( {{\text{Fe}}_{3} {\text{O}}_{4} } \right)$$
2: Second nanoparticle ($${\text{Al}}_{2} {\text{O}}_{3}$$)
3: Third nanoparticle ($${\text{TiO}}_{2}$$)
4: Forth nanoparticles (Cu)

## Greek symbols

$$\alpha$$: Thermal diffusivity (m^2^/s)
$\varphi$: Equivalent nanoparticles volume fraction
$\varphi_{1}$: Equivalent first nanoparticles volume fraction
$\eta$: Independent similarity variable
$\varphi_{2}$: Equivalent first nanoparticles volume fraction
